# Changes in Physiological and Pathological Behaviours Produced by Deep Microelectrode Implantation Surgery in Rats: A Temporal Analysis

**DOI:** 10.1155/2020/4385706

**Published:** 2020-03-09

**Authors:** Gustavo A. Chiprés-Tinajero, Miguel A. Núñez-Ochoa, Laura Medina-Ceja

**Affiliations:** Laboratory of Neurophysiology, Department of Cellular and Molecular Biology, CUCBA, University of Guadalajara, Mexico

## Abstract

Physiological behaviours such as the sleep-wake cycle and exploratory behaviours are important parameters in intact and sham-operated animals and are usually thought to be unaffected by experimental protocols in which neurosurgery is performed. However, there is insufficient evidence in the literature on the behavioural and cognitive effects observed after deep microelectrode implantation surgery in animal models of neurological diseases. Similarly, in studies that utilize animal models of neurological diseases, the impact of surgery on the pathological phenomena being studied is often minimized. Based on these considerations, we performed a temporal analysis of the effects of deep microelectrode implantation surgery in the hippocampus of rats on quiet wakefulness, sleep, and exploratory activity and the pathological behaviours such as convulsive seizures according to the Racine scale. Male Wistar rats (210-300 g) were used and grouped in sham and epileptic animals. Single doses of pilocarpine hydrochloride (2.4 mg/2 *μ*l; i.c.v.) were administered to the animals to generate spontaneous and recurrent seizures. Deep microelectrode implantation surgeries in both groups and analysis of Fast ripples were performed. Physiological and pathological behaviours were recorded through direct video monitoring of animals (24/7). Our principal findings showed that in epileptic animals, one of the main behaviours affected by surgery is sleep; as a consequence of this behavioural change, a decrease in exploratory activity was also found as well as the mean time spent daily in seizures of scale 4 and the number of seizure events of scales 4 and 5 was increased after surgery. No significant correlations between the occurrence of FR and seizure events of scale 4 (rho 0.63, *p* value 0.25) or 5 (rho -0.7, *p* value 0.18) were observed. In conclusion, microelectrode implantation surgeries modified some physiological and pathological behaviours; therefore, it is important to consider this fact when it is working with animal models.

## 1. Introduction

Physiological behaviours such as the sleep-wake cycle and exploratory behaviours are important parameters in intact and sham-operated animals and are usually thought to be unaffected by experimental protocols in which neurosurgery is performed. However, there is insufficient evidence in the literature on the behavioural and cognitive effects observed after deep microelectrode implantation surgery in animal models of neurological diseases compared with studies carried out in patients with various brain pathologies [1, 2]. In addition, most existing studies emphasize the morphological and molecular findings after surgery, such as inflammatory response, blood brain barrier disruption, or even fine motor deficit in control versus experimental animals without regard to physiological behaviours [3–9]. Similarly, in studies that utilize animal models of neurological diseases, the impact of surgery on the pathological phenomena being studied is often minimized. One example is research involving seizures and epilepsy models in which electrophysiological parameters, such as high-frequency oscillations, which are known to be associated with the disease are studied without regard to the possible effects of surgery. Moreover, these surgeries typically involve specific brain areas, such as the hippocampus and cortex; these areas contain circuits that are associated with the cognitive process through theta and gamma rhythms and ripple events and with specific physiological behaviours, such as the sleep-wake cycle [10–12]. Most of these surgeries involve the implantation of electrodes or microelectrodes into the brain for the purpose of recording electrical signals and/or the implantation of guide cannulas that are used to insert dialysis probes or fine needles for drug administration [10–13]. Based on these considerations, we performed a temporal analysis of the effects of deep microelectrode implantation surgery in the hippocampus of sham and epileptic rats on physiological and pathological behaviours. The physiological behaviours studied were quiet wakefulness, sleep, and exploratory activity, and the pathological behaviours studied were convulsive seizures according to the Racine scale. These behaviours were assessed over a period extending from 15 days before to 15 days after the surgery. In addition, (a) the relationship between the latency of the first spontaneous seizures and the time that lasted rats in Racine scales 3, 4, and 5 per day before and after surgery and (b) the correlation between the number of fast ripples (FR) recorded and the number and severity of seizure events on the same day were analysed.

## 2. Materials and Methods

### 2.1. Animals and Administration of Pilocarpine

Male Wistar rats weighing 210-300 g (*n* = 14) were used. The animals were housed in a room with a 12-hour light and dark cycle at a temperature of 24-27°C and were permitted free movement and access to water and food. The handling and maintenance of the animals were approved by the local animal care committee and conducted in accordance with the Norms for Research in Health Matters (Mexican Official Norms NOM 062-ZOO-1999 and NOM-033-ZOO-1995). The rats were divided into two groups: sham animals (*n* = 7) and epileptic animals (*n* = 7).

Single doses of pilocarpine hydrochloride (2.4 mg/2 *μ*l; Sigma-Aldrich, USA) were administered intracerebroventricularly (i.c.v.) to the animals in the epileptic group to generate a model of induction of spontaneous and recurrent seizures [14]. For this purpose, the animals were anaesthetized with oxygen-isoflurane prior to pilocarpine injection into the right lateral ventricle (AP -4.5 mm, ML -5.2 mm, and DV -7 mm from bregma) by means of a needle connected to an injection pump attached to the stereotactic frame (Stoelting Co., IL, USA). After pilocarpine administration, the behaviour of the animals was observed until status epilepticus (SE) based on the criteria of the Racine scale [15]. SE was indicated if the animals presented seizure-like events of scale 4 or 5 (Table 1). The SE was stopped after 90 minutes by systemic injection of diazepam (5-10 mg/kg, i.p.) to ensure the animals' survival. Following this procedure, the animals were subjected to video monitoring 24/7 to detect spontaneous and recurrent seizures until subsequent microelectrode implantation surgery.

### 2.2. Microelectrode Implantation Surgery

Both sham and epileptic rats were implanted with deep microelectrodes. The epileptic animals received the surgery 15 days after the first seizure was observed. The animals were anaesthetized with isoflurane in oxygen, and an array of 10 tungsten microelectrodes 60 *μ*m in diameter was implanted into the right hippocampal region. The 5 bipolar electrodes in the array were separated from each other by a distance of 500 *μ*m. The reported dorsoventral coordinate is the deepest relative to bregma. The electrodes were arranged as follows. One bipolar microelectrode was placed in CA3 (AP: -5.04 mm, ML: -4.5 mm, and DV: -6.5 mm), and two bipolar microelectrodes for DG were placed in the molecular (AP: -6.48 mm, ML: -4.6 mm, and DV: -5.6 mm) and polymorphic (AP: -6.48 mm, ML: -4.6 mm, and DV: -4.6 mm) layers. Granular layer recording was achieved with a derivation between tips, and DG full recording was obtained through the derivation between the nearest tip to the surface and the deepest tip inside DG. Two bipolar microelectrodes for CA1 were placed in the pyramidal (AP: -6.72 mm, ML: -5.8 mm, and DV: -4.8 mm) and radial (AP: -6.72 mm, ML: -5.8 mm, and DV: -5.6 mm) layers, and the 2 surface electrodes above bregma were considered as ground and indifferent. To confirm the correct position of microelectrodes, the animals' brains were removed and cut in coronal sections (50 *μ*m thick) in order to proceed with an immunohistochemistry directed to neurons (NeuN, data not showed).

### 2.3. EEG Recordings

After allowing a three-day period for recovery from the deep microelectrode implantation surgery, the animals in both groups (sham and epileptic animals) were recorded under free movement conditions for 90 minutes on days 1, 2, 3, 7, and 14. We used AcqKnowledge Data Acquisition software 4.0 as a user interface (BIOPAC Systems, USA) with an MP150 (BIOPAC Systems, CA, USA) as an analogue-to-digital converter for the recordings, which were conducted via polygraph (Model 7D, Grass Technologies, RI, USA) at a bandwidth of 0.1 to 5 kHz and sampling at 2.5 kHz per channel (7 channels) with 12-bit precision using an iMac A1048 (Apple, USA).

### 2.4. Detection of Fast Ripples and Correlation with Seizure Severity

The inclusion characteristics for FR selection in the present work were as follows: (1) FR were selected visually, (2) possible FR that had linear noise > 15 *μ*V or peak-to-peak amplitude greater than 150 *μ*V were eliminated, and (3) the recordings that passed the threshold were subjected to continuous wavelet transformation to ensure that the frequency event was temporally delimited and to eliminate the presence of 60 Hz or harmonics in the signal.

The analysis included the band from 250 to 600 Hz and was normalized to the highest wavelet energy coefficient in the recording channels; if the frequencies of possible FR coincided with the expected frequencies, the events were evaluated and classified as FR.

Signal and data analysis was realized offline using personalized programs written in MATLAB (MathWorks, Inc., USA), Python (License of Python Software Foundation, USA), or R (R Foundation for Statistical Computing, GNU General Public License, USA). A total of 346 events classified as FR were obtained in the different registration areas; these were correlated with different scales to determine the linear relationship of FR occurrence to the severity of seizures and indirectly relate it to the effects of surgery. For this purpose, we performed a Pearson correlation between the mean FR registered per day of EEG recording and the mean number of FR events of scales 4 and 5 that occurred on the same day of EEG recording (1, 2, 3, 7, and 14 days after surgery).

### 2.5. Analysis of Physiological Behaviours

The physiological behaviours of the animals in the sham and epileptic groups were analysed according to episodes of sleep, wakefulness, and exploration. The analysis was performed over a period extending from 15 days before to 15 days after the deep electrode implantation surgery. Physiological behaviour was recorded through direct video monitoring of animals (24/7). Episodes in which the rat remained lying down with closed eyes were recorded as sleep; in quiet wakefulness, rats remained motionless but with open eyes. Scratching behaviours and episodes of movement related to eating or drinking were not included in the analysis. During exploratory episodes, the animals were in constant motion, continuously sniffing in the box with the presence of vibrissa movements, grooming, and raising. The purpose of this analysis was to compare the physiological behaviours of sham and epileptic rats before and after surgery.

### 2.6. Analysis of Convulsive Behaviour

To analyse the animals' convulsive behaviour, the epileptic rats were subjected to continuous observation by video monitoring (24/7), and the latency to the appearance of the first spontaneous seizure of scale 4 or 5 on the Racine scale was recorded. The ranking of convulsive behaviour according to the Racine scale is as follows: scale 1, movement of lips and tongue, vibrissae movement, and salivation; scale 2, head clonus and eye clonus; scale 3, forelimb clonus, “wet dog shakes”; scale 4, raising of the forelimbs with clonic convulsions; and scale 5, raising of the forelimbs with clonic convulsions and loss of posture. Once the first seizure was presented, the behaviours of scales 3, 4, and 5 and their duration were quantified. This analysis was performed over a period extending from 15 days before to 15 days after the surgical implantation of deep electrodes.

### 2.7. Statistical Analysis

Comparisons between groups and surgery effects (pre- vs. postsurgery and intragroup) were performed using Student's *t*-test after Q-Q plots and the Shapiro-Wilk test for normality had confirmed that the *t*-test was suitable. Statistical significance was defined as obtaining *p* values < 0.05. Pearson correlation was performed to correlate FR occurrence with seizure severity.

## 3. Results

**Figure 1:**
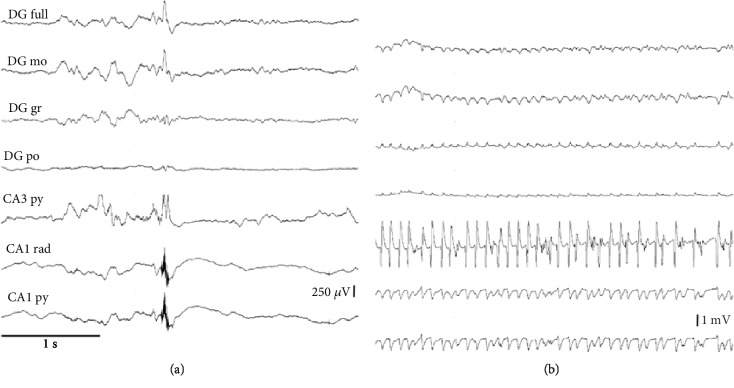
Representative EEG recordings from animals in the epileptic group. (a) Representative FR observed through the microelectrodes placed in the CA1 region. (b) Representative epileptiform activity observed in CA3. The seizure pattern was characterized by spike-wave activity characteristic of seizures of intensity 5 on the Racine scale. Abbreviations: EEG: electroencephalogram; FR: fast ripples; CA: cornu ammonis; mo: molecular cell layer; gr: granule cell layer; po: polymorphic cell layer; py: pyramidal cell layer; rad: radiatum cell layer.

In the analysis of deep EEG recordings obtained from the epileptic group, a total of 346 FR events were observed in the registration areas. When these were correlated with different scales to determine whether there was a linear relationship between FR occurrence and the severity of seizures and indirectly relate it to the effects of surgery, no significant correlations between the occurrence of FR and events of scale 4 (rho 0.63, *p* value 0.25) or 5 (rho -0.7, *p* value 0.18) were observed (Figure 1).

**Figure 2:**
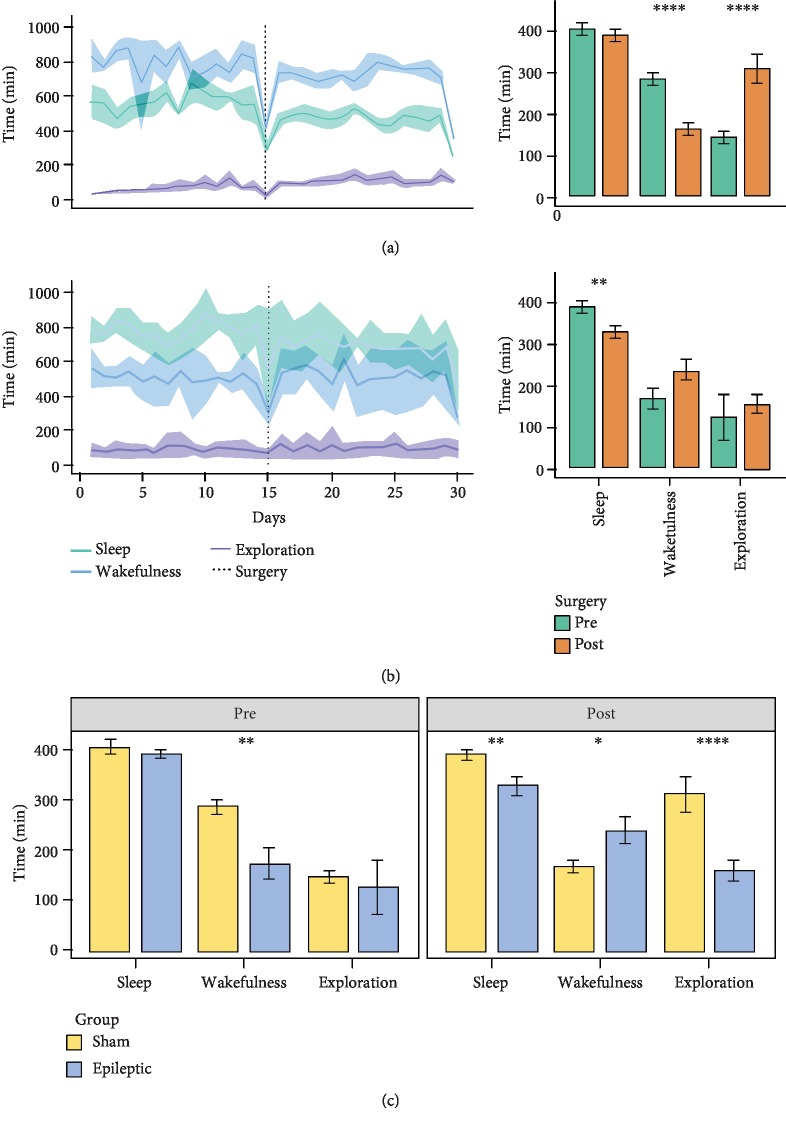
Analysis of physiological behaviours before and after microelectrode implantation surgery. (a) Line plot showing changes in the physiological behaviours of sham animals over time. The solid line represents the sample mean, and the shadowed region indicates the 95% confidence interval; comparisons between pre- and postsurgery effects in sham animals are shown at the right. (b) As in (a), but for animals in the epileptic group. (c) Differences in the physiological behaviours of the sham and epileptic groups related to surgery. The *t*-test was used for each comparison (^∗^*p* < 0.05, ^∗∗^*p* < 0.01, ^∗∗∗^*p* < 0.001, and ^∗∗∗∗^*p* < 0.0001).

Epileptic animals showed a significant decrease in sleep time after surgery (*t*-test, *p* = 0.009), but their periods of quiet wakefulness and exploratory behaviours were not modified by surgery (Figure 2(a)). On the other hand, a reduction in the duration of the quiet wakefulness period was observed in animals in the sham group after surgery (*t*-test, *p* < 0.00001), and the time spent by these animals in exploratory behaviour increased (*t*-test, *p* < 0.00001) (Figure 2(b)). Comparison of the physiological behaviours of epileptic and sham animals showed a significant increase in quiet wakefulness time in the sham group before surgery (*t*-test, *p* = 0.0081) and an increase in sleep and exploratory behaviour time after surgery (*t*-test, *p* = 0.0086 and *p* < 0.00001, respectively) (Figure 2(c)).

**Figure 3:**
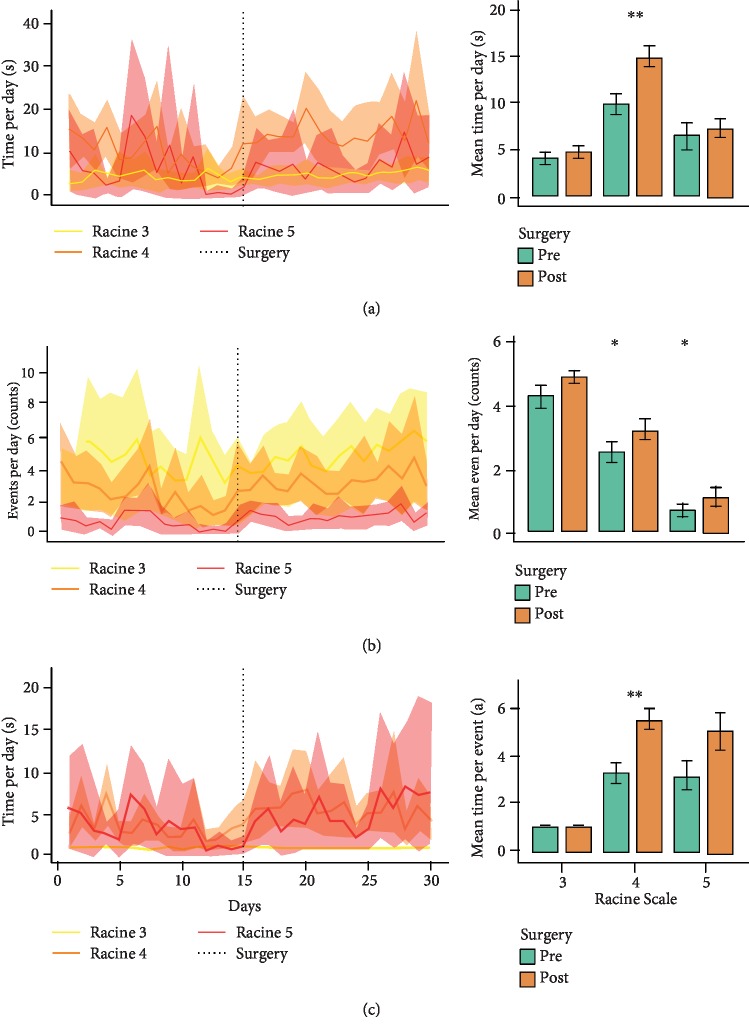
Analysis of the severity of convulsive behaviour according to the Racine scale before and after microelectrode implantation surgery. The line plots show the changes in the severity of convulsive behaviour as a function of time. The solid line represents the sample mean, and the shadowed region indicates the 95% confidence interval; the graphs at the right show comparisons of seizure severity (Racine scales 3, 4, and 5) before and after surgery. The *t*-test was used for all comparisons (^∗^*p* < 0.05, ^∗∗^*p* < 0.01, ^∗∗∗^*p* < 0.001, and ^∗∗∗∗^*p* < 0.0001).

**Figure 4:**
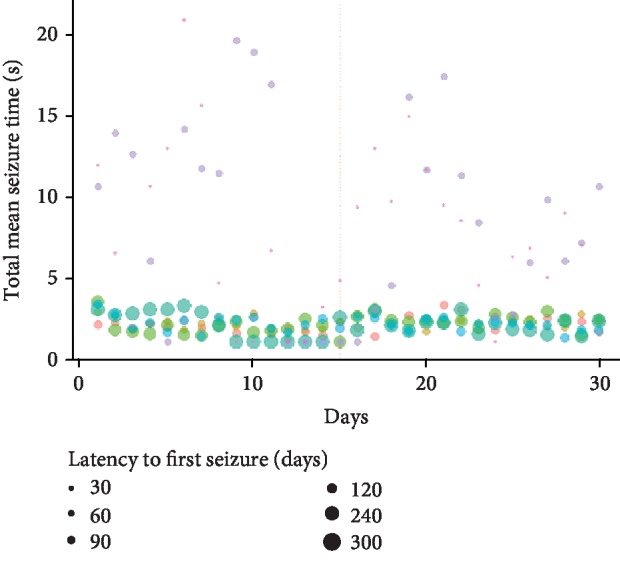
Relationship of the temporal course of seizure event duration to latency to the first spontaneous and recurrent seizure. Each different color point represents an individual rat from the epileptic group (*n* = 7); the size of the point is proportional to the latency time to the first spontaneous seizure (time elapsed between pilocarpine injection and the first spontaneous and recurrent seizure observed); surgery is indicated by a dotted line. Note that all the rats with latencies over 60 days are grouped around 2-4 seconds per event, while rats with latencies less than 60 days show high variability. This could be the result of a more consolidated epileptogenic process in the animals that showed longer latency. Additionally, the fact that the grouping pattern remains unchanged after the surgery shows that the surgery did not affect the epileptogenic process.

In addition, the results showed that scale 4 events persisted for a long time after surgery (*t*-test, *p* = 0.0012); an increased number of events of scales 4 and 5 were also observed (*t*-test, *p* = 0.02 and *p* = 0.01, respectively) (Figures 3(a) and 3(b)). However, the ratio between the duration of events and the number of events of each scale showed an increase only for seizures of scale 4 (Figure 3(c)) after surgery (*t*-test, *p* = 0.004). When we analysed the mean duration per event of the total seizures observed during each day of the period extending from 15 days before to 15 days after surgery in each animal, we found that surgery did not modify the daily pattern of seizures observed in the animals (Figure 4). The rats with short latency to the first spontaneous seizure (≤60 days) showed high variability in the duration of seizure events of scale 4 on the Racine scale both before and after surgery. In contrast, animals with long latency (≥90 days) showed a stable pattern in this parameter before and after surgery (Figure 4).

## 4. Discussion

We found no difference in the average sleep time of the sham and epileptic groups during the 15 days prior to surgery; however, it should be noted that despite spending the same amount of time sleeping per day as the sham animals, the epileptic rats engaged in more sleep periods. This is consistent with the phenomenon of sleep fragmentation in which increased light sleep and decreased sleep efficiency, deep sleep, and rapid eye movement (REM) sleep occur; this phenomena has been described both in humans [16] and in animal models [17–19]. Likewise, the epileptic rats spent less time in quiet wakefulness and exploratory behaviour compared with the sham group. Alterations similar to these are observed in patients with temporal lobe epilepsy and are involved in learning and memory [18, 20, 21]. However, 15 days after implantation surgery, decreased sleep time and exploratory behaviour were observed in the epileptic rats. This could be evidence of decreased sleep quality in these animals [19, 21–23] and may indicate that various diurnal symptoms such as excessive sleepiness or attention disorders were present in the animals during the time of quiet wakefulness, resulting in the decrease in exploratory behaviour observed in the epileptic animals. This effect in place could be similar to a phenomenon that has been observed in patients with TLE [18, 19, 23]. However, one limitation of our study was not comparing intact versus epileptic animals without implantation surgery. However, this result is consistent with the results of a previous study in which the implantation of deep electrodes for EEG recording modified the circadian cycle in rats with epileptic seizures, first decreasing their motor activity and subsequently increasing it [24]. An advantage of our study is that behaviours were classified as quiet wakefulness, exploration, and sleep and not merely as activity related to the light-dark cycle. We were able to rule out the possibility that the behavioural effects produced by surgery on the epileptic group were caused by the pathogenic process itself because the same behaviours were analysed over the 15-day period prior to surgery. In contrast, in the sham group, a significant increase in the average time spent exploring and sleeping as well as a decrease in the amount of time spent in quiet wakefulness was observed after surgery. Physiological behaviours of this type are closely related to oscillatory activities in the hippocampus, such as gamma and theta rhythms [24, 25], as well as to various types of learning, such as spatial learning, planning [26, 27], and memory [28, 29]. In addition, there is evidence that cortical and hippocampal lesions that cause neuronal loss and extensive damage affect diverse behaviours [30]. Therefore, the injury generated by surgery could alter the rhythmic control of hippocampal activity and, at the same time, affect related behaviours through inflammatory responses and the death of distinct inhibitory interneurons in the hilus of the dentate gyrus [24]. The effect of this altered inhibition on pyramidal cells in the hippocampus was also observed in other studies in which spatial and nonspatial learning and memory tests were performed during epileptogenesis and TLE [20, 26, 27, 31–33].

In the same way, surgery increased the average duration of seizure events of scale 4 and the number of seizure events of scales 4 and 5. Although postsurgical modifications in the intensity of seizure events according to the Racine scale have not been described in the literature, a modification of the circadian cycle in animals subjected to surgery has been described [24]. Our results are consistent with the results of other studies in which an increase in the number and duration of seizures was observed after surgical electrode implantation [17–19]. However, the observed increase in the number of seizure events was not accompanied by an increase in the average amount of time during which the rat experienced seizures of scale 5; seizures of scale 3 were not altered during the 30-day period (the number of events and the duration of seizures of scale 3 remained at a ratio of approximately 1 : 1). These results may be because it is easy to differentiate behaviours related to scale 3, such as shaking, because they persist for a longer time than physiological behaviours, such as scratching, grooming, and behaviours related to discomfort of the animal. The fact that there are technical difficulties associated with determining the loss of posture of epileptic animals during the video monitoring and thereby distinguishing between seizures of scales 4 and 5 could explain why events of scale 4 were the only class of events that showed a significant increase in duration after surgery.

To determine whether the observed change in seizure severity was due to surgery or was a direct effect of epileptogenesis, an intragroup analysis was performed in which the relationships between the latency to the first spontaneous seizure, the total duration of seizures of scales 3, 4, and 5, and the total number of seizure events per day per animal were assessed. The results showed a high variability in animals with latency < 60 days compared with animals with latencies of ≥90 days. The epileptic animals with long latencies showed 4 s of seizures per day, very similar to the parameter of average time per event of scale 4, and this pattern remained after surgery and did not influence [34] or modify epileptogenesis; in addition, these animals displayed a better established pathological process in which there was less variability over time. Therefore, the changes observed inside the group of epileptic animals after surgery could be due to the intrinsic relationship between sleep and epilepsy [35, 36] as well as to the impact of the sleep-wake cycle on the development of seizures. This is consistent with evidence found in human patients [37, 38].

Finally, no correlation between the severity of seizures according to the Racine scale and FR activity was found in the present study, probably because it was difficult to time the EEG recordings to coincide with the animals' seizures. This is a technical limitation that can be improved by extending the time during which EEG recordings are made. In addition, it is difficult to determine the effect of surgery implantation on FR because they can only be observed and analysed through deep EEG electrodes; therefore, the effect of implantation is limited even if we try to determine it indirectly by severity of scales before and after implantation surgery. Although we cannot compare our results with similar data in the literature, Bragin and coworkers [33, 39, 40] demonstrated a direct relationship between early occurrence of FR and FR occurrence with early spontaneous and recurrent seizures in a TLE model induced by kainic acid.

## 5. Conclusions

We conclude that in epileptic animals, one of the main behaviours affected by microelectrode implantation surgery is sleep; as a consequence of this behavioural change, a decrease in exploratory activity was also found. In addition, the mean time spent daily in seizures of scale 4 was increased in epileptic rats that received microelectrode implantation surgery, and an increase in the number of seizure events of scales 4 and 5 was observed in these animals after surgery. In contrast, the animals in the sham group showed a reduction in quiet wakefulness and an increase in sleep and exploratory activity after surgery. Another important observation made in our study is that the latency to the first seizure and its variability showed a stable pattern in animals in which the latency was greater than 90 days. This sheds light on the development of the convulsive process in which cellular, molecular, and electrophysiological changes must occur to permit the establishment and the generalized course of epilepsy. In the 24/7 analysis of video monitoring for 30 days, we observed that for seizures of scale 5 to occur, scale 4 seizures are necessary, and it was in events of this scale 4 in which changes in number and time were observed; furthermore, these changes persisted during the 15 days following surgery. Finally, no significant correlations between the occurrence of FR and seizure events of different scales were observed.

## Figures and Tables

**Table 1:** Latency to status epilepticus (SE) in minutes and weight of rats. Rats were injected with pilocarpine (2.4 mg/2 *μ*l, i.c.v.) until SE was observed.

Rat	Minutes	Weight
1	90	228
2	135	233
3	90	205
4	40	297
5	120	253
6	75	211
7	45	215

## Data Availability

The data used to support the findings of this study are available from the corresponding author upon request.
